# The gut microbiome in the Lebanese population and its alterations in metastatic colorectal cancer: benchmarking and application

**DOI:** 10.1186/s12866-026-05325-9

**Published:** 2026-06-29

**Authors:** Sally Temraz, Farah J Nassar, Pierre Bertrand, Razan Al Tartir, Maria Mezher, Ruba Hadla, Zahraa S. Msheik, Ghada Chamandi, Vincent Cahais, Cyrille Cuenin, Ayman Al Shoukari, Ali Shamseddine, Hibeh Shatila, Farah Naja, Yinan Zheng, Lifang Hou, Rihab Nasr, Akram Ghantous

**Affiliations:** 1 Naef K. Basile Cancer Center, Department of Internal Medicine, American University of Beirut Medical Center, Beirut, Lebanon; 2 International Agency for Research on Cancer, Lyon, France; 3 Department of Radiology, Children’s Hospital of Philadelphia, Philadelphia, PA USA; 4 IT Medical Center Applications Department, American University of Beirut, Beirut, Lebanon; 5 Department of Anatomy, Cell Biology and Physiological Sciences, American University of Beirut, Beirut, Lebanon; 6 Department of Emergency Medicine, American University of Beirut Medical Center, Beirut, Lebanon; 7 Department of Nutrition Sciences, College of Health Sciences, QU Health, Qatar University, Doha, P.O. Box 2713, Doha, Qatar; 8 Department of Clinical Nutrition and Dietetics, College of Health Sciences, Research Institute of Medical and Health Sciences (RIMHS), University of Sharjah, Sharjah, United Arab Emirates; 9 Faculty of Agriculture and Food Sciences, American University of Beirut, Beirut, Lebanon; 10 Department of Preventive Medicine, Northwestern University Feinberg School of Medicine, Chicago, IL USA

**Keywords:** Colorectal cancer, Gut, Microbiome, Lebanese population, 16S rRNA sequencing, Ddysbiosis, Benchmarking

## Abstract

**Background:**

Colorectal cancer (CRC) is a rising health concern in Lebanon and across the Arab world, concomitant with dietary and lifestyle transitions. The gut microbiome may contribute to CRC progression and treatment response, and it remains understudied in Arab populations. This pilot study investigates gut microbiome dynamics in a cohort of stage IV CRC patients and cancer-free individuals from Lebanon, benchmarking computational tools for 16S rRNA sequencing and analyzing microbial and functional shifts associated with cancer development and therapy.

**Methods:**

Stool samples were collected from cancer-free individuals (*n* = 32) and newly diagnosed stage IV CRC patients before therapy (*n* = 17). A subset of CRC patients (*n* = 10) provided follow-up samples 3–6 months after therapy. 16S rRNA microbial profiling was conducted using the Illumina MiSeq platform. Computational tools were benchmarked using a mock community, with a defined microbial composition. Microbiome and functional analyses included diversity metrics, differential abundance testing, and pathway prediction, with adjustments for age and sex, followed by sensitivity analyses accounting for antibiotic use and immunotherapy.

**Results:**

Among the evaluated computational tools, USEARCH was selected for downstream CRC microbiome analyses based on its performance. CRC patients exhibited progressive dysbiosis, characterised by distinct beta diversity profiles, reduced alpha diversity and a declining Firmicutes/Bacteroidota ratio from cancer-free controls to baseline CRC and post-therapy samples. Differential abundance analysis identified taxa associated with CRC development and therapy response, including *Fusobacterium*, *Muribaculaceae*,* Intestinimonas*,* Intestinimonas butyriciproducens* and *Lactobacillus fermentum*. Notably, some potentially beneficial taxa increased progressively across the disease and treatment continuum, suggesting that therapy may promote specific favorable microorganisms while coinciding with broader microbiome dysregulation. Functional pathway analysis revealed widespread alterations in microbial functional capacity, including potential therapy-associated shifts.

**Conclusion:**

This study represents a pioneering effort in gut microbiome profiling of Lebanese population with advanced CRC, establishing a molecular reference map during a period of rising CRC incidence and dietary transition. Computational benchmarking using a mock community supported the robustness of the analytical workflow. The results may facilitate the discovery of microbial signatures associated with CRC development and therapy response, providing insights into cancer prevention and clinical intervention.

**Supplementary Information:**

The online version contains supplementary material available at https://doi.org/10.1186/s12866-026-05325-9.

## Introduction

Colorectal cancer (CRC) ranks as the third most common cancer and remains the second leading cause of cancer-related deaths worldwide [1]. In Lebanon, CRC is the fourth most frequently diagnosed malignancy, with similarly high mortality rates [2]. Across the Arab world, CRC incidence has risen over recent decades, likely driven by changes in lifestyle and dietary habits [3]. The transition from traditional diets to Westernized food consumption patterns, including an increased reliance on processed and ready-made meals, has been linked to shifts in gut microbiota composition and potential impacts on carcinogenesis [4]. Notably, in Lebanon, more than two-thirds of CRC patients are diagnosed at advanced stages, which indicates a significant problem with late-stage cancer detection in the country [5]. Despite advances in chemotherapy and targeted therapies, stage IV CRC patients continue to experience poor clinical outcome, with a five-year survival rate of only 14%, rendering them terminally ill [6]. Considerable inter-individual variability in therapy response and toxicity further highlights the need for biomarkers that can support personalized therapeutic strategies.

Growing evidence suggests that the gut microbiome plays a pivotal role in modulating cancer treatment outcomes, influencing both therapy efficacy and toxicity. Microbial diversity and composition can impact the effectiveness of chemotherapy and immunotherapy, with certain bacterial taxa enhancing drug metabolism and immune response [7]. Conversely, microbiome dysbiosis, which is characterized by reduced microbial diversity and an imbalance of pathogenic bacteria, has been linked to poor response to immune checkpoint inhibitors and increased chemotherapy-related toxicity [8]. These findings generated interest in microbiome-targeted interventions such as probiotic supplementation, dietary interventions, and fecal microbiota transplantation to optimize microbiome health and improve therapeutic efficacy [9]. However, most microbiome studies in CRC have been conducted in Western populations, while data from Arab populations remain limited despite major regional dietary and epidemiological transitions. Moreover, longitudinal microbiome dynamics during CRC therapy are still insufficiently characterized.

Microbiome studies are also highly influenced by analytical methodology. Although 16S rRNA sequencing remains a widely used and cost-effective approach for microbial profiling [10], several bioinformatics tools have been designed for microbiome sequencing analysis, beginning with Mothur and QIIME, followed by USEARCH, which later evolved with the UPARSE algorithm and UNOISE [11–15]. However, comparative assessments of these pipelines remain limited. Standardizing analytical approaches is therefore important to improve reproducibility and ensure reliable interpretation of microbiome-associated clinical findings.

**Fig. 1:**
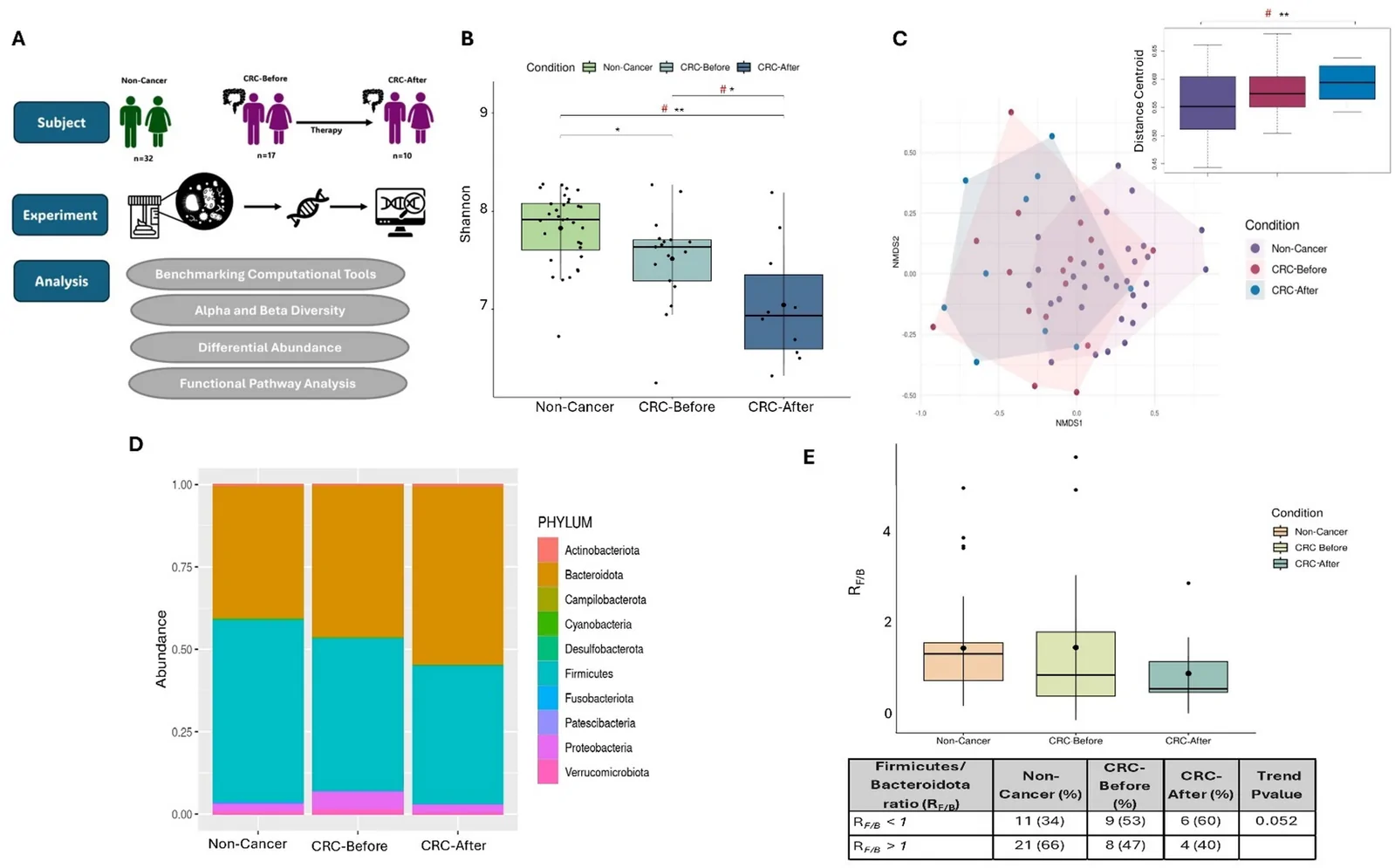
Microbiome Diversity across three conditions of the CRC cohort and the Non-Cancer subjects.**A** Summary of the microbiome experimental and the analytical workflows. **B** Alpha diversity using Shannon Index of the microbiota between the three conditions in CRC cohort and Non-Cancer subjects. Whiskers represent the minimum and the maximum, while the top, the bottom, and the band in the box represent the first and third quartile and the median, respectively. Each data point in the box plot represents one patient, and the large black dot in the middle of each boxplot represents the mean. Shapiro-Wilk normality test demonstrated deviation from normality, so the non-parametric Kruskal-Wallis followed by Post-Hoc Dunn’s test was performed for the statistical comparison between the conditions: * FDR < 0.05, ** FDR < 0.01 and red # FDR for linear mixed effect model accounting for paired samples and with adjustment for age and sex. Further statistical analysis and details were provided in the supplementary methods and Supplementary Table 3. **C** Beta diversity for the gut microbiome derived from fecal samples of CRC cohort and Non-Cancer subjects. The non-metric multidimensional scaling (NMDS) plot is based on the Bray–Curtis dissimilarity distances. Beta dispersion across the groups was presented as boxplot and differences in community composition between groups were assessed using PERMANOVA. ** FDR < 0.01 and red # p-value for regression model with adjustment for age and sex. **D** Dominant phyla relative abundance for the gut microbiome derived from fecal samples of CRC cohort and Non-Cancer subjects. **E** Firmicutes/Bacteroidota ratio (R_F/B_) across the three conditions shown in a table and a boxplot format with borderline trend p-value using Cochrane-Armitage test for trend analysis. **B**-**E** All graphs include subjects grouped into three conditions: Non-Cancer (*n* = 32), CRC-Before (*n* = 17) meaning CRC-Before therapy and CRC-After (*n* = 10) meaning CRC patient after 3–6 months of therapy. Microbiome data was analyzed using USEARCH ASV method

In this study, we first evaluated commonly used microbiome analysis pipelines to ensure methodological precision in microbiome analysis. Using the top-performing workflow, we characterized the gut microbiome of healthy Lebanese individuals, offering baseline data from a population undergoing rapid dietary and lifestyle transitions. In addition, we investigated a cohort of treatment naïve stage IV CRC cohort to address the limited microbiome data available for advanced stage disease in underrepresented Arab populations (Fig. 1A). We finally longitudinally assessed the microbiome shifts before and after therapy, providing insights into therapy associated microbial shifts and potential guidance for future personalized treatment strategies of CRC patients. Ultimately, our findings highlight the importance of analytical pipeline selection, provide region-specific microbiome data, and support the integration of microbiome research into precision oncology for CRC patients.

## Materials and methods

### Literature mining for microbiome sequencing analysis tools

A literature search was done to identify computational tools for 16S rRNA gene sequencing analysis published until May 2022. PubMed database was used as a search engine with the Boolean operators that combined several “Medical Subject Headings” terms (MeSH). The first stage was literature mining focused on microbiome analysis independent of disease or target tissue. This led to the following syntax: “metagenomics [MeSH] AND bioinformatics” which yielded 2,664 publications. Publications were evaluated using the following eligibility criteria: (1) written in English, (2) being original, and (3) focusing on analysis tools. To avoid missing relevant articles, the references cited in the prioritized articles were screened for additional publications, review articles were examined for analysis tools, and a complementary literature search was performed using the Google Scholar engine with the query: “bioinformatics AND 16S microbiome”. Literature search was repeated by adding a colorectal biological relevance to the microbiome analysis tools, using the following syntax on PubMed: “metagenomics [MeSH] AND (Colorectal Cancer OR Gastrointestinal Microbiome)”. This led to the identification of the articles focusing on CRC and microbiome and the gut mock microbiome data which was downloaded and used in the downstream analysis.

### Datasets

#### Mock community

A mock community, Mock1_130417, with a known composition of 18 genera, was downloaded from the Mothur website [16]. This mock community was employed to test quality control and optimize data analysis pipelines, with sequencing performed using the Illumina MiSeq platform covering a 250 bp (bp) length of reads.

#### Colorectal cancer cohort and non-cancer subjects

##### Patient recruitment and biospecimen collection

This prospective IRB-approved cohort was conducted at the American University of Beirut Medical Center (AUBMC), a tertiary healthcare center in Lebanon. It involved the recruitment of non-cancer participants and newly diagnosed treatment-naïve stage IV CRC patients (according to TNM staging) between 2018 and 2022. All participants were adults over 18 years old, with no history of immune related or inflammatory diseases. A survey assessing antibiotics use prior to specimen collection was also conducted. Non-Cancer subjects (*n* = 32) provided stool samples only once, while CRC patients provided stool samples prior to their initial therapy (*n* = 17) and again after 3–6 months of therapy (*n* = 10). All patients received chemotherapy, except one who received immunotherapy. Participants self-collected stool samples using sterile, leak-proof containers and single-use collection tools. Detailed instructions were provided on aseptic techniques and contamination avoidance. Samples collected at the clinical site were promptly stored at -80 °C. For home collections, samples were kept in freezers at − 20 °C and transported to the clinic, where they were immediately transferred to − 80 °C for long-term storage and subsequent analysis.

##### Microbiome sequencing

DNA was extracted from the stool samples using QIAamp DNA Stool Mini Kit (Qiagen, Germany) according to the manufacturer’s instructions. Microbiome profiling was performed at the International Agency for Research on Cancer (IARC) by deep-sequencing of variable V3 and V4 regions of the 16S rRNA gene using paired end reads (Supplementary Table 1). A 589 bp amplicon covering V3-V4 regions was generated and subjected to library preparation using Illumina sequencing adapters and dual-index barcodes according to guidelines from 16S Metagenomic Sequencing Library Preparation. Sequencing was performed using the Illumina MiSeq benchtop instrument. Approximately 0.22 million reads were generated per sample, exceeding the minimum read recommendations of Illumina protocols. Quality filtering was performed using a minimum Phred quality score threshold of 30, and sequences shorter than 30 bp were trimmed using Trim Galore (v 0.6.10).

### Benchmarking computational tools for microbiome analysis using mock data

Mock community (i.e. with known microbial composition) was used to optimize microbiome analysis using three computational tools (Mothur v1.40.4, USEARCH v11 and DADA2 v1.22) (details are provided in the Supplementary methods and Supplementary Fig. 1). Performance metrics were used to assess the efficacy of the computational tools. These metrics include: sensitivity (true positive rate), specificity (true negative rate), True Positive (TP, representing the number of correctly identified genera), False Positive (FP, representing the number of spurious genera predicted by the pipeline), True Negative (TN, representing the number of genera excluding those assigned to the mock, calculated as the total number of unique genera in the RDP v18 reference file (estimated 3240) minus the number of predicted genera) and False Negative (FN, representing the number of expected genera not identified by the pipeline). According to these definitions, the sensitivity and specificity were calculated using the following formulas: Sensitivity = TP / (TP + FN) and Specificity = TN / (TN + FP).

### Microbiome analysis in colorectal cancer cohort and non-cancer subjects

#### Analysis of microbiome abundance data

Microbiome analysis was performed using our workflow available on the GitHub repository (https://github.com/IARCbioinfo/wsearch-nf.git), implemented with USEARCH v11 and VSEARCH v2.21.1_linux_x86_64, with taxonomic assignment based on the SILVA v138 reference database. When lower-level taxonomic assignment was not possible, the lowest confidently assigned taxonomic label was retained. Archaea domain, Chloroplast order and the mitochondria family were filtered out from the dataset. Variations in age, sex and body mass index (BMI) across the three conditions (“Non-Cancer” for non-cancer participants, “CRC-Before” for CRC patients before therapy, “CRC-After” for CRC patients after therapy) were tested using Kruskal-Wallis test followed by post hoc Dunn’s test for pairwise testing with false discovery rate (FDR) adjustment (Table 2). Phyloseq R package v1.48.0 was used to visualize sample taxonomy. The abundance of each taxonomic cluster was normalized in each sample by transforming abundance to percentage to facilitate comparison across samples. Shannon, Inverse Simpson and Chao1 indices were used to measure alpha diversity. Shapiro-wilk test showed non-normal distributions of alpha diversity indices across the different conditions; hence, the non-parametric Kruskal-Wallis test followed by post hoc Dunn’s test for pairwise testing was applied. To account for pairing and adjust for age and sex, linear mixed effect model was performed (details provided in Supplementary Methods). Bray-Curtis dissimilarity distances represented in non-metric multidimensional scaling (NMDS) plots were used to assess the beta diversity. PERMANOVA was used to assess differences in beta diversity among the conditions with and without adjustment for sex and age. Firmicutes/Bacteriodota ratio (R_F/B_) was calculated for each condition, and the frequencies of R_F/B_ > 1 and < 1 were summarized. Frequencies across ordered conditions were compared using a one-sided Cochran-Armitage test to assess the observed increasing trend of the proportion of samples exhibiting R_F/B_ <1.

Microbiome abundance data were filtered with a minimum total read count of 5000 counts per sample, a minimum total abundance threshold of 10 counts per taxon and the presence of each taxon in at least 5 samples [17, 18]. Global testing, multiple pairwise comparison (Dunnet’s test) and pattern analysis of the microbiome abundance were performed using Analysis of Compositions of Microbiomes with Bias Correction 2 (ANCOM-BC2) package v2.6.0, a bias-corrected method developed for compositional microbiome data analysis. Differential abundance was analyzed according to the sample condition (cancer-free and CRC before and after therapy) with and without adjustment for age and sex. Adjusted p-values (q-values) were calculated using the Holm–Bonferroni method which is more stringent than FDR-based approaches as it limits false positive discoveries at the taxon level. For the multiple pairwise comparison, CRC-Before was set as the reference group. For the pattern analysis, the Non-Cancer group was set as the reference. A robustness analysis evaluating the pseudo-count addition was also performed. Circo plots were generated using circlize R package v0.4.16.

#### Functional analysis

PICRUSt2 was used to predict functional abundances using Python v3.12 [19] with a maximum nearest sequenced taxon index (NSTI) cut-off of 2. Principal Component Analysis (PCA) analysis of pathways across the three conditions was visualized using ggpicrust2 package v2.5.2. Linear Models for Differential Analysis (LinDA v0.2.0) was applied on the PICRUSt2 output to identify Kyoto Encyclopedia of Genes and Genomes (KEGG) Orthology (KO) pathways that are significantly different across conditions. Results were filtered for log2 fold change threshold ($\:\ge\:$ |2|) and adjusted p-values (< 0.05), calculated using FDR correction method.

## Results

### Benchmarking of computational tools for microbiome targeted sequencing

Computational tools used for microbiome 16S rRNA sequencing analysis were evaluated in two phases based on (1) literature mining and (2) analysis of a mock dataset with known microbiome composition. Supplementary Table 2 summarizes the comparative evaluation of the various tools. In terms of biological resolution, DADA2, which is the most recent computational R-based method, provides higher biological resolution than the traditional operational taxonomic unit (OTU) clustering because it differentiates strains more accurately using the amplicon sequence variant (ASV) denoising, with the trade-off of generating unusually high number of clusters [15]. Another reason for DADA2’s high resolution is its learned error model that identifies sequencing errors. USEARCH has comparable biological resolution compared to DADA2 and is the most optimized tool due to its implementation of Bash as compared to the other R-based packages. Regarding denoising methods, USEARCH and QIIME2 are so far the only tools capable of performing both OTU clustering and ASV denoising. In terms of computational efficiency, USEARCH performs the fastest clustering (approximately 2 min/sample), though DADA2 and QIIME2 are also relatively fast (approximately 4 min/sample), while Mothur is substantially slower (approximately 60 min/sample). In terms of documentation quality, algorithms of USEARCH and QIIME2 are well explained, which facilitates the use of the commands and the modification of parameters, whereas the algorithms of Mothur and DADA2 are less comprehensively documented (Supplementary Table 2). Based on this conceptual evaluation, Mothur seems to be the least performing tool as compared to the other tools. USEARCH, QIIME and DADA2 have relatively similar performances, each with differential strengths and weaknesses in various criteria. QIIME2 supports both ASV-based analysis through integrated DADA2 denoising workflows and OTU-based clustering using the VSEARCH pipeline, an open-source alternative to USEARCH. Therefore, QIIME2 was excluded from the downstream mock community analysis to minimize methodological redundancy.

The performance of USEARCH, DADA2 and Mothur was evaluated using the 16S rRNA sequencing of a mock dataset with a known composition of 18 genera (Table 1). Firstly, Mothur and USEARCH had a similar number of predicted clusters (122 and 120 clusters, respectively), while DADA2 predicted less clusters. Similar results were obtained upon application to the dataset of the CRC cohort and Non-Cancer subjects. Even though USEARCH predicted fewer genera than Mothur, USEARCH correctly identified all 18 genera present in the mock community, whereas Mothur identified only 14 (Table 1). Secondly, focusing on the ASV algorithms, the number of clusters identified by USEARCH was higher than that by DADA2. This pattern was also replicated in the subsequent application on CRC cohort and Non-Cancer subjects. USEARCH identified 17 of the 18 genera compared to 15 identified by DADA2, indicating that USEARCH provides less spurious clusters than the other tools, whether relying on the OTU or ASV method. Within USEARCH, the ASV workflow generated more clusters than the OTU workflow across all datasets analyzed. USEARCH OTU approach correctly identified all 18 expected genera of the mock community, while ASV workflow missed one genus (Table 1).

**Table 1:** Performance metrics of 16S rRNA microbiome analysis tools predicting 18 genera in a mock dataset

	Mothur	USEARCH		DADA2
	OTU	OTU	ASV	ASV
Predicted Number of Clusters	122	120	173	61
Predicted Number of Genera	72	41	25	39
Correctly Identified Genera	14	18	17	15
True Positive (TP)	14	18	17	15
False Positive (FP)	72–14 = 58	41 − 18 = 23	25 − 17 = 8	39 − 15 = 24
True Negative (TN) *	3222-72 = 3150	3222-41 = 3181	3222-25 = 3197	3222-39 = 3183
False Negative (FN)	18 − 14 = 4	18–18 = 0	18 − 17 = 1	18 − 15 = 3
Specificity (%)	98.2	99.3	**99.8**	99.3
Sensitivity (%)	77.7	**100**	94.4	83.3

*The value of 3222 was derived from the total number of genera in the RDP v18 reference file (*n* = 3240) minus the number of expected genera in the mock community (*n*= 18)

Bold font signifies highest specificity and sensitivity

All the tools had high specificity (98.2–99.8%), indicating good performance in correctly identifying true negatives. In terms of sensitivity, Mothur showed the least (77.7%), DADA2 achieved 83.3%, while USEARCH ASV and OTU approach achieved 94.4% and 100%, respectively. Hence, USEARCH was selected for downstream analyses because of its highest sensitivity, high specificity, and based on methodological considerations, including its support for both OTU- and ASV-based workflows, ability to provide comparable clustering outputs under both approaches, lowest computing time per sample, and consistent performance across datasets.

### Microbiome analysis of the colorectal cancer cohort and non-cancer subjects

#### Microbiome diversity

Variations in microbiome composition were investigated across three participant groups: 32 Non-Cancer, 17 CRC-Before and 10 CRC-After. No significant differences were observed across the participant groups for sex and BMI (Table 2). Age was significantly different between Non-Cancer and each of CRC-Before and CRC-After (Table 2).

**Table 2:** Characteristics of the Non-Cancer subjects and the CRC cohort.

Characteristics	Non-Cancer (NC)	CRC-Before (CB)	CRC-After (CA)	Kruskal-Wallis (*p*-Value)	Post Hoc Dunn’s test (FDR)	
*N*	32	17	10			
*Age*,* median(Q1-Q3)*	42.6 (34.5–49.5)	56 (43–69)	56.5 (49.5–68.2)	**1.00E-03**	**CB vs. NC**:	**6.04E-03**
					**CA vs. NC**:	**6.04E-03**
					CA vs. CB:	7.04E-01
*Sex*,* N(%)*	Male: 18 (56) Female: 14 (44)	Male: 6 (35) Female: 11 (65)	Male: 3 (30) Female 7(70)	2.55E-01	CB vs. NC:	4.16E-01
					CA vs. NC:	4.16E-01
					CA vs. CB:	1.00E + 00
*BMI*,* median (Q1-Q3)*	28.6 (25.7–31.4)	28.5 (23.6–32.7)	27.8 (26–29.7)	9.35E-01	CB vs. NC:	8.68E-01
					CA vs. NC:	8.68E-01
					CA vs. CB:	8.68E-01

Kruskal–Wallis test was used to compare age, sex, and BMI across the three conditions, followed by post hoc Dunn’s test with FDR adjustment for pairwise comparisons. Q1: first quartile; Q3: third quartile; *FDR *false discovery rate, *NC *Non-Cancer, *CB *CRC-Before, *CA *CRC-After

Alpha diversity variations were assessed across age, sex, and BMI categories using Shannon, Inverse Simpson and Chao1 indices. Age showed limited and method-dependent associations with alpha diversity, in both ASV- and OTU-based analyses (Supplementary Tables 3a & 3e). These associations generally indicated a trend toward lower diversity with increasing age; however, they were no longer significant in a sensitivity analysis excluding samples exposed to antibiotics or immunotherapy (Supplementary Table 3c). Sex showed even weaker associations, with a significant difference observed only for ASV-based Chao1 richness, which was lower in females than in males; this association was not retained in the sensitivity analysis (Supplementary Tables 3a, 3c & 3e). No significant associations were observed between BMI and any alpha diversity metric in either the ASV-Main or sensitivity analyses (Supplementary Tables 3a, 3c & 3e).

Alpha diversity differed across participant groups, with CRC patients, particularly those in the CRC-After group, generally exhibiting lower diversity than Non-Cancer controls (Fig. 1B, Supplementary Figs. 2 A-B, Supplementary Table 3b). Shannon, Inverse Simpson and Chao1 diversities were significantly lower in CRC-After relative to Non-Cancer controls. Lower Shannon and Chao1 diversities were also observed in CRC-Before compared with Non-Cancer controls; however, these differences were attenuated after accounting for paired samples and adjusting for age and sex, while the reduced diversity in CRC-After remained significant (Supplementary Table 3b). Although no significant difference was detected between the two CRC groups in the ASV-Main analysis, the paired longitudinal analysis revealed significantly lower Shannon diversity in CRC-After than in matched CRC-Before patients (Supplementary Table 3b). Sensitivity analyses yielded broadly consistent results, with only minor differences across diversity metrics (Supplementary Table 3d). Likewise, the complementary OTU-based analysis reproduced the overall pattern of reduced alpha diversity in CRC, particularly in CRC-After, with Chao1 richness remaining significant after adjustment (Supplementary Table 3f, Supplementary Figs. 3 A-C).

Beta diversity analyses corroborated the alpha diversity findings, with CRC-After patients clustering away from the Non-Cancer subjects, while the CRC-Before patients clustered in between. Group differences were significant in both unadjusted and adjusted PERMANOVA analyses (Fig. 1C & Supplementary Fig. 2C). Similar patterns were observed in the sensitivity analyses (PERMONOVA, *p* = 0.001) and the complementary OTU based analyses (Supplementary Fig. 3D) .

#### Microbiome structural composition and differential abundance

Identical phyla compositions were obtained using both ASV- (Fig. 1D, Supplementary Fig. 2D) or OTU-based workflows (Supplementary Figs. 3E-F). Analysis of the gut microbiome in the CRC cohort and the Non-Cancer subjects revealed that two phyla, Bacteroidota and Firmicutes, were predominant in all samples (Fig. 1B, Supplementary Fig. 2D). An increased proportion of Bacteroidota and a decreased proportion of Firmicutes were detected in CRC-Before as compared to Non-Cancer, and these proportional differences became more pronounced in CRC-After (Figs. 1D-E). The average Firmicutes/Bacteroidota ratio (R_F/B_) in Non-Cancer was greater than one, and this ratio decreased in CRC-Before and further diminished in CRC-After to less than 1 (Fig. 1E). To confirm these observations, the proportions of samples having R_F/B_ < 1 were compared across the three participant groups. An increasing trend (*p* = 0.05) was observed of this proportion from Non-Cancer subjects (34%) to CRC-Before (53%) and CRC-After (60%) (Fig. 1E).

**Fig. 2:**
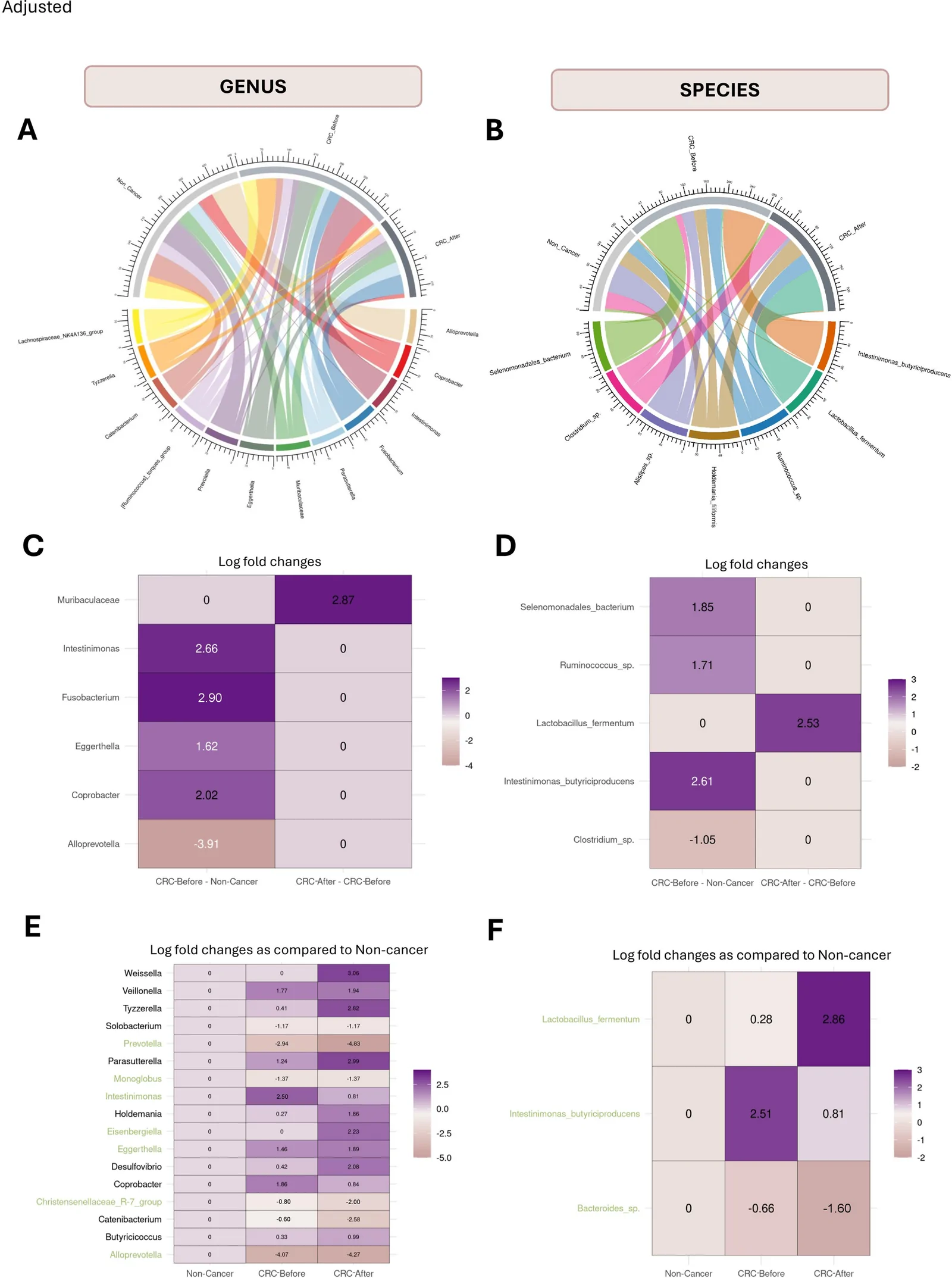
Differential microbiome abundance across three conditions of the CRC cohort and the Non-Cancer subjects with adjustment for age and sex. **A**,** B** Circo plots showing the relative abundance of significantly differentially abundant genera and species, respectively, identified by the global test across the three conditions. **C**, **D** Significant genera and species, respectively, identified by multiple pairwise comparisons against CRC-Before group (Dunnett’s type of test). The columns denote the specific comparisons: CRC-Before versus Non-Cancer and CRC-After versus CRC-Before. Each cell color represents significant changes in absolute abundance: purple for increased abundance and pink for reduced abundance. The text within each cell represents the log fold-change value relative to the reference group with the white color denoting passing the robustness analysis for the pseudo-count addition while the black color denoting not passing it. **E**, **F** Significant genera and species, respectively, identified by pattern analysis using Non-Cancer as the reference group. Monotonically increasing and decreasing trends were evaluated across the three conditions, with Non-Cancer as the reference. Each cell color represents abundance change: purple for increased abundance and pink for reduced abundance. Each cell contains log fold-change relative to the reference group. Genera/Species names represented in green denote passing the robustness analysis for the pseudo-count addition. **A**-**F** All graphs include subjects grouped into three conditions: Non-Cancer (*n* = 32), CRC-Before (*n* = 17) meaning CRC patients before therapy and CRC-After (*n* = 10) meaning CRC patients after 3–6 months of therapy. Statistical tests were done using ANCOM-BC2 with sex and age adjustment and multiple testing correction for (C-F) was done using the Holm–Bonferroni method

Microbiome differential abundance was analysed across the three participant groups (Non-Cancer, CRC-Before, CRC-After) using ANCOM-BC2 through global tests (Figs. 2A-B), Dunnett’s comparisons (Figs. 2C-D), and pattern analyses (Figs. 2E-F). Analyses were conducted with and without adjustment for age and sex, as well as in sensitivity analyses excluding samples exposed to immunotherapy or antibiotics (Fig. 2, Supplementary Fig. 5, Supplementary Tables 4–6). To identify the most robust microbial signatures, we focused on taxa that were consistently significant across adjustment models and sensitivity analyses.

In the global test, significant differences across the three groups were consistently observed for the phylum Fusobacteriota, the family *Fusobacteriaceae*, and the genera *Fusobacterium*, *Intestinimonas*, *Prevotella*, *Parasutterella* and *Muribaculaceae* (Fig. 2A, Supplementary Table 4a). Significant species-level associations included *Ruminococcus sp.*,* Alistipes sp.*,* Intestinimonas butyriciproducens* and *Lactobacillus fermentum* (Fig. 2B, Supplementary Tables 4a-c).

Dunnet’s comparisons identified consistent significant differences between Non-Cancer and CRC-Before, primarily for the genera *Fusobacterium* and *Intestinimonas* and the species *Intestinimonas butyriciproducens* (Figs. 2C-D, Supplementary Tables 5a-c). Additional differences between CRC-After and CRC-Before were observed for *Muribaculaceae* and *Lactobacillus fermentum*, although these findings were not consistently significant across all analyses.

Pattern analysis was performed to identify taxa showing progressive changes across Non-Cancer, CRC-Before, and CRC-After groups. Several taxa exhibited consistent trends across the three groups (Figs. 2E-F, Supplementary Tables 6a-c). Across all analyses, the family *Fusobacteriaceae* was enriched mainly in CRC-Before; whereas, *Muribaculaceae* increased only in CRC-After relative to Non-Cancer controls (Supplementary Table 6a). *Parasutterella* and *Veillonella* showed a progressive increase from Non-Cancer to CRC-Before and CRC-After; in contrast, *Prevotella* showed progressive depletion across CRC groups, with the largest reduction observed in CRC-After (Fig. 2E). *Intestinimonas* (Fig. 2E) and *Intestinimonas butyriciproducens* (Fig. 2F) markedly increased in CRC-Before but showed reduced effect sizes in CRC-After, while *Lactobacillus fermentum* showed a stronger increase in CRC-After (Fig. 2F).

#### Functional pathway analysis

**Fig. 3:**
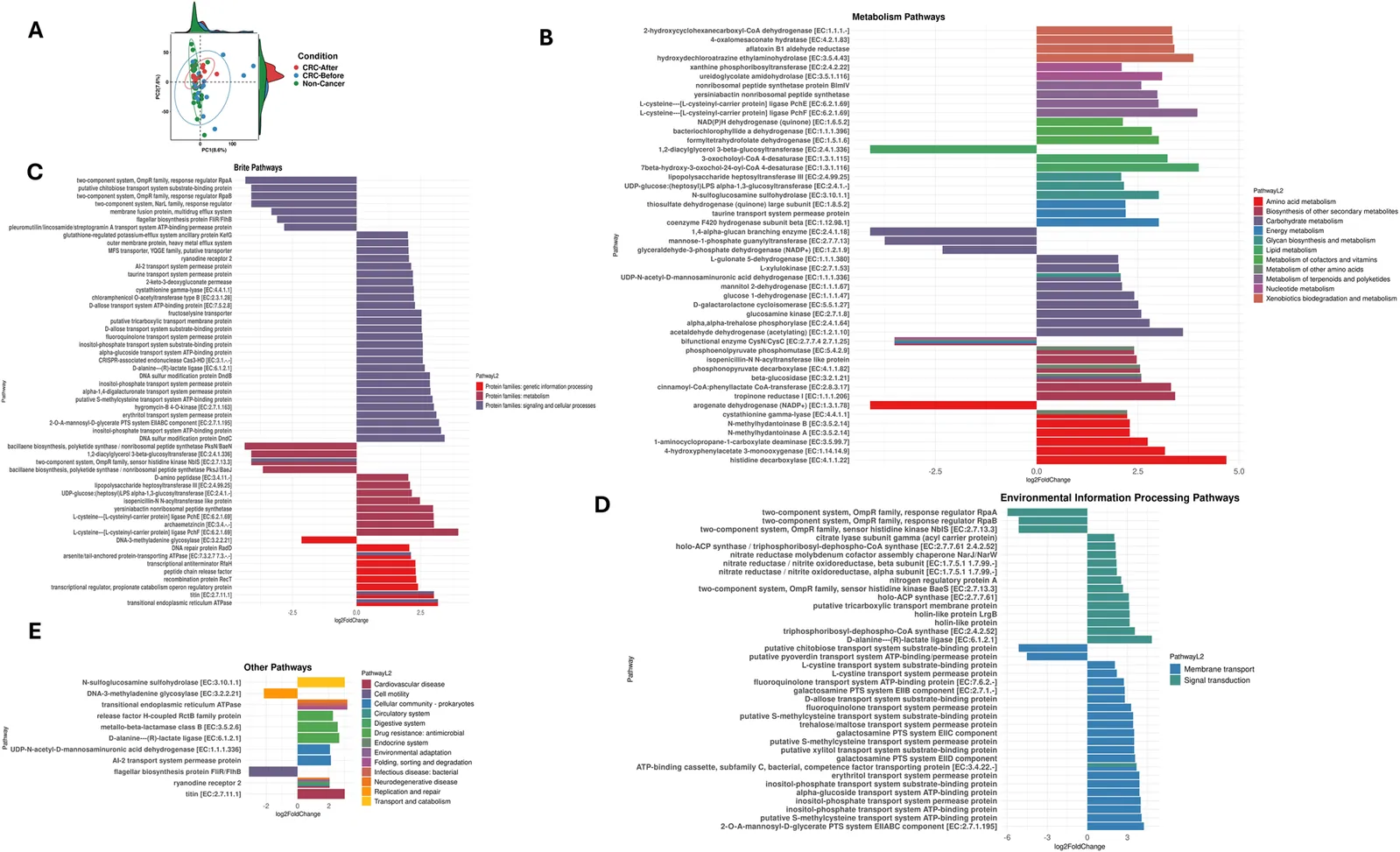
Microbiome functional analysis in CRC-Before compared to Non-Cancer subjects.**A** Principal Component Analysis (PCA) plot on functional pathway abundance data for three conditions (Non-Cancer, CRC-Before and CRC-After). **B**-**E** Bar plots representing the log Fold Change for the significant microbiota pathways in CRC-Before compared to Non-Cancer subjects grouped into (**B**) Metabolism pathways, **C** Brite pathways, **D** Environmental Information Processing pathways and **E** Other pathways (Pathways are color coded by Pathway L2 groups and filtered by log Fold Change $\:\ge\:$ |2|, FDR adjusted p-value < 0.05). **A** includes subjects grouped into three conditions: Non-Cancer (*n* = 32), CRC-Before (*n* = 17) meaning CRC patients before therapy and CRC-After (*n* = 10) meaning CRC patients after 3–6 months of therapy. (B-D) All graphs include subjects grouped into two conditions: Non-Cancer (*n* = 32) and CRC-Before (*n* = 17)

PCA of predicted functional pathway abundances revealed distinct functional profiles across the three participant groups, with greater separation between CRC patients and non-cancer controls than between CRC-Before and CRC-After (Fig. 3A).

**Fig. 4:**
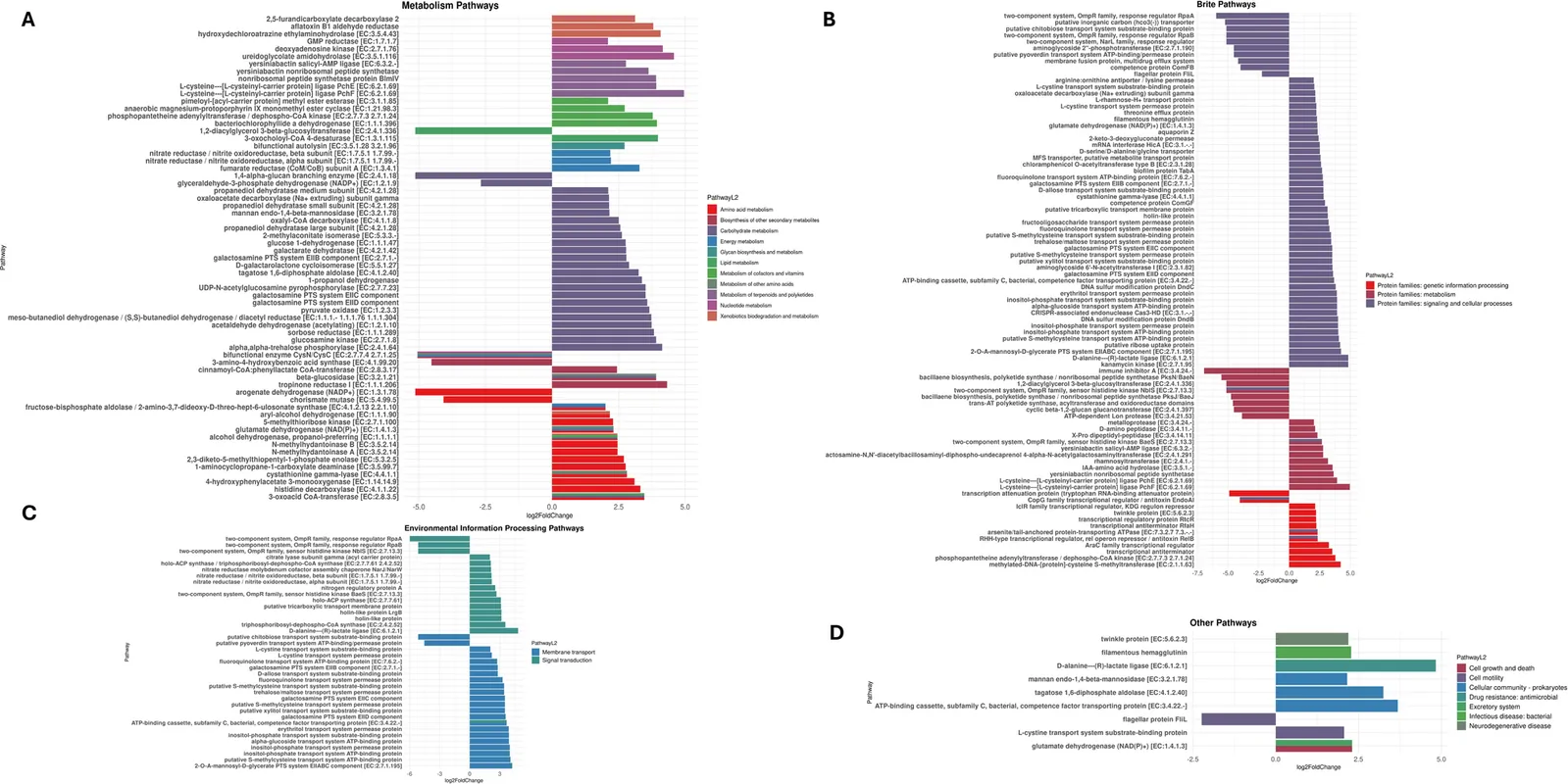
Microbiome functional analysis in CRC-After compared to Non-Cancer subjects.**A**-**D** Bar plots representing the log Fold Change for the significant microbiota pathways in CRC-After compared to Non-Cancer subjects grouped into (**A**) Metabolism pathways, **B** Brite pathways, **C** Environmental Information Processing pathways, and **D** Other pathways (Pathways are color coded by Pathway L2 groups and filtered by log Fold Change $\:\ge\:$ |2|, FDR adjusted p-value < 0.05). (A-D) All graphs include subjects grouped into two conditions: Non-Cancer (*n* = 32) and CRC-After (*n* = 10) meaning CRC patients after 3–6 months of therapy

Compared to Non-Cancer controls, 122 KO pathways were differentially enriched in CRC-Before (FDR < 0.05, Figs. 3B-E, Supplementary Table 7a) and 175 in CRC-After (FDR < 0.05, Fig. 4 & Supplementary Table 7b). These pathways primarily mapped to metabolic functions (Figs. 3B and 4A), environmental information processing (Figs. 3D and 4C), and Brite hierarchies, particularly transporters enzymes, antimicrobial resistance, transcription factors, prokaryotic defense system and DNA repair and recombination proteins (Figs. 3C and 4B).

Most dysregulated pathways were enriched in CRC relative to Non-Cancer controls (83–85%), with 22% of the pathways (*n* = 68) being shared between CRC-Before vs. Non-Cancer and CRC-After vs. Non-Cancer comparisons. When compared to Non-Cancer, several functional categories showed a greater increase in CRC-After than CRC-Before, including amino acid and carbohydrate metabolism, transporters, two-component system, transcription factors, prokaryotic defense system pathways and nitrogen metabolism. In contrast, lipopolysaccharide biosynthesis, DNA repair and recombination proteins and sulfur metabolism were more prominently enriched in CRC-Before. These patterns remained consistent after excluding samples exposed to immunotherapy or antibiotics, although fewer pathways remained statistically significant because of the reduced sample size (Supplementary Tables 7a1-b1).

Direct comparison of CRC-After and CRC-Before samples identified no significantly different pathways after FDR adjustment (Supplementary Table 7c). However, exploratory analysis based on nominal p-values identified 60 pathways enriched in CRC-After, mainly related to antimicrobial resistance and stress adaptation (Supplementary Table 7c). Similar trends were observed following exclusion of antibiotic- and immunotherapy-exposed samples and were further supported by paired longitudinal analyses of matched pre- and post-therapy samples (Supplementary Tables 7c1-c2).

## Discussion

The gut microbiome plays an essential role in human development, immunity and metabolism. Dysbiosis of the gut microbiome has been increasingly associated with several diseases, including CRC. In this study, we first evaluated commonly used computational pipelines for 16S rRNA microbiome analysis to assess their impact on microbial profiling outcomes and improve methodological consistency. We then implemented these pipelines to characterize the gut microbiome of healthy Lebanese individuals and treatment-naïve metastatic CRC patients from an underrepresented Arab population. Finally, through longitudinal sampling before and after therapy, we investigated potential therapy-associated microbiome alterations.

Literature mining identified major computational tools for microbiome analysis, namely Mothur, USEARCH, DADA2 and QIIME/QIIME2, together with two analytical strategies, OTU clustering and ASV denoising. The tools were first conceptually evaluated according to several methodological and computational criteria, leading to the elimination of QIIME/QIIME2 in the downstream analyses because it integrates workflows already presented by other selected tools. Both the conceptual evaluation and the benchmarking convened that Mothur showed lower performance, leading to its elimination from further downstream analyses. USEARCH and DADA2 had similar specificity, but USEARCH exhibited a higher sensitivity, reaching 100% under the OTU workflow. Although DADA2 provided higher biological resolution through ASV-based denoising, it did not necessarily improve recovery of expected taxa in the mock community. Additionally, USEARCH offered the advantage of supporting both OTU and ASV methods. Therefore, USEARCH was selected for downstream CRC microbiome analyses based on its performance, computational efficiency and methodological flexibility. Overall, our benchmarking results were broadly consistent with previous comparative evaluations of 16S rRNA bioinformatics pipelines, including Prodan et al., who confirmed strong overall performance of USEARCH relative to Mothur and comparable to DADA2, supporting the reliability of the pipeline selection used in the present study [20].

In terms of the overall microbiome variation, the alpha diversity decreased in CRC patients relative to Non-Cancer subjects, especially after therapy. This association remained across crude and adjusted statistical models controlling for age and sex as well as in the sensitivity analysis. Similar results were obtained using beta diversity metrics, highlighting that CRC development is associated with microbiome dysbiosis, and that anticancer therapy further deregulates the microbiome in the CRC patients as reported in other cancer types. Non-Hodgkin’s lymphoma patients who received myeloablative conditioning regimen revealed severe compositional and functional imbalance in the gut microbial community associated with chemotherapy-induced gastrointestinal mucositis [21].

Bacteriodota and Firmicutes, the major members of the gut microbiota, were the most predominant phyla present in both Non-Cancer and CRC patients. Interestingly, the proportion of Bacteriodota increased and that of Firmicutes decreased in treatment-naïve stage IV CRC patients relative to Non-Cancer. This is in accordance with previous findings from a North American [22] and an Asian population [23]. The Enterotoxigenic *Bacteroides fragilis* (from the Bacteriodota phyla) were also reported to be significantly higher in CRC patients especially those in late stages (III/IV) compared to control samples [24, 25]. The Firmicutes phyla is known for producing butyrate with anti-inflammatory and anti-tumor effects [26]. Indeed, the balance between the predominant Bacteriodota and Firmicutes phyla, represented by the R_F/B_ ratio, has been proposed as a marker of microbiome dysbiosis and has been associated with disease progression, although its reliability and universality remain debated [27]. A large gut microbiome cohort study on 1,135 Dutch adult participants reported higher abundances of Firmicutes (63.7%) than Bacteriodota (8.1%) [28], highlighting that the R_F/B_ is usually high in Non-Cancer, as observed in our results. Our results demonstrated that this ratio further decreased in CRC patients, especially after therapy, suggesting that anticancer therapy may contribute to persistent microbiome dysbiosis rather than restoring microbial composition back to that of Non-Cancer controls, consistent with the patterns observed in alpha and beta diversities. This is in contrast to tumor ablation therapy in CRC patients which can restore the microbiome back to that of Non-Cancer subjects [23]. In vivo mice study demonstrated that 5-fluorouracil (5-FU), a major chemotherapeutic drug given to CRC patients, diminished the bacterial community richness and diversity, leading to the relatively lower abundance of Firmicutes and decreased R_F/B_ in feces and cecum contents [29]. Causality of the associations was reinforced by fecal transplant from healthy mice which prevented body weight loss and colon shortening of 5-FU-treated mice. These findings were complemented by bacterial cell models showing that key changes in bacterial community dynamics occur under chemotherapeutic stress and that the bacteria interact together in drug detoxification to promote microbiota resilience [30]. This further advocates for the need for probiotic development that may provide gut-specific protection to patients undergoing cancer treatment.

Distinct microbial signatures were associated with CRC status and therapy. Consistent with previous CRC microbiome studies, members of the phylum Fusobacteriota and the family *Fusobacteriaceae*, including the genus *Fusobacterium*, were enriched predominantly in CRC-Before, supporting their established association with CRC initiation and development [31, 32]. Similarly, *Intestinimonas* was enriched in CRC-Before dominated by *I. butyriciproducens.* This species was reported to be elevated in young CRC patients in a meta-analysis of fecal metagenomics data from multi-cohorts [33], overrepresented in CRC in gut mucosal tissues from a Malaysian population [34] and positively associated with CRC in Italian and Swedish populations, as per the Human Gut Microbiome Atlas with data from 20 different countries [35].

In contrast, *Muribaculaceae* showed a significant increase in CRC-After, which coincides with its protective role in the gut and cooperative interactions with probiotics, such as *Bifidobacterium* and *Lactobacillus* [36, 37]. Indeed, *Lactobacillus fermentum* displayed a progressive increase from Non-Cancer through CRC-Before to CRC-After, with post therapy enrichment similar to the *Muribaculaceae*. This goes in line with its probiotic biotherapeutic potential in CRC by inhibiting the proliferation of CRC cell lines through secreted short chain fatty acids (SCFAs) [38–40] or in combination of 5-FU while protecting normal cells from the 5-FU cytotoxic effects [41]. Importantly, the enrichment of these potentially beneficial taxa after therapy should not be interpreted as restoration of a healthy microbiome. Rather, despite the increase in selected protective microorganisms, CRC-After samples remained characterized by reduced diversity and a microbial composition distinct from Non-Cancer, suggesting that therapy may simultaneously promote specific beneficial taxa while contributing to broader microbiome dysbiosis. Overall, the observed microbial shifts followed an anticipated progression from Non-Cancer to CRC-Before and CRC-After, consistent with the microbiome variation indices. Certain alterations persisted even post-therapy, likely due to residual tumor tissues in those stage IV CRC patients or the effect of therapy. The persistence of these associations after adjustment for age and sex, as well as in the sensitivity analyses, indicates that the observed microbial patterns were not driven solely by potential confounding or therapy-related outliers.

Predicted functional pathway analysis revealed substantial alterations in microbial functional capacity in CRC patients compared with Non-Cancer controls, consistent with the observed results of microbiome variation. Compared with Non-Cancer, CRC-After exhibited similar altered pathways to CRC-Before related to metabolism, transporters, two-component system, transcription factors and prokaryotic defense system pathways. In contrast, relatively limited variation was observed between CRC-Before and CRC-After groups, suggesting either persistence of CRC-associated functional dysbiosis following therapy or limited statistical power to detect differences because of the small sample size. Exploratory analysis comparing CRC-After to CRC-Before, based on nominal p-values, identified pathways associated with antimicrobial resistance and stress adaptation. Similar trends were observed in sensitivity and paired analyses; however these findings should be interpreted cautiously as no pathways remained significant after FDR correction. Nevertheless, they may provide hypothesis-generating insights for future studies. One possible explanation for the enrichment of antimicrobial resistance pathways is the selective pressure imposed by chemotherapy. Notably, ten chemotherapeutic drugs including oxaliptatin, a standard CRC chemotherapy, have been shown to accelerate the emergence of antibiotic-resistant mutants from bacterial commensals [42]. This observation warrants further investigation, especially given a rise in antibiotic resistance in cancer patients, for whom infections remain the second leading cause of death [43].

Despite the limitations of this pilot study, a number of strengths can be highlighted. Although the relatively small sample size reduced the statistical power of the analyses and limited the robustness of intergroup comparisons, the findings provide an important exploratory foundation for future investigations. Benchmarking of bioinformatics workflows using both literature-based evaluation and a mock community with known microbial composition allowed the application of an optimized pipeline on a unique understudied population. Furthermore, evidence of microbiome dysbiosis across the three participant groups was triangulated using multiple approaches, including ASV- and OTU- based analyses, adjustment for confounders, sensitivity analyses and alternative statistical tools, thus, increasing the robustness of the observed microbial signatures. The inclusion of stage IV CRC patients represents a strength, as stage-specific microbiome studies in advanced metastatic CRC remain limited. Such a design minimizes variability associated with tumor stage and enables a more focused investigation of microbiome alterations linked to metastatic disease and therapeutic response. Nevertheless, residual confounding factors, including dietary habits, smoking status, medication use, socioeconomic background, and other lifestyle-related exposures may have influenced microbiome composition and could not be fully controlled in this pilot study. Such variables, as well as survival data are currently being collected as part of this evolving work. Given the rising evidence that the gut microbiome can influence therapeutic efficacy, chemoresistance, and therapy-related toxicity [44], longitudinal follow-up studies are warranted to investigate the prognostic and therapeutic implications of microbiome dysbiosis in advanced CRC.

In conclusion, this study represents the first characterization of the gut microbiome in Lebanese patient with stage IV CRC and cancer-free subjects. By leveraging next-generation sequencing, state-of -the-art bioinformatics analyses, and benchmarked computational tools, we established a preliminary molecular map of the gut microbiome in an understudied population experiencing rising CRC incidence and obesity alongside rapid dietary transition toward a Westernized lifestyle [2, 45]. Our findings identified microbial signatures associated with CRC and highlighted potential therapy-related microbiome alterations, including the prevalence of biotherapeutic species along with potential predicted functional changes related to antibiotic resistance. While these observations require further validation, they provide insights for the discovery of microbiome markers of CRC development, anticancer therapy, and prognosis, ultimately supporting the development of more personalized patient management and clinical intervention to improve patient outcomes.

## Supplementary Information

Supplementary Material 1: Supplementary Methods [20, 46–49].

Supplementary Material 2: Supplementary Figure 1. Pipelines for microbiome 16S rRNA sequencing analysis using Mothur, USEARCH and DADA2 packages.

Supplementary Material 3: Supplementary Figure 2. Microbiome Diversity across three conditions of the CRC cohort and the Non-Cancer subjects using ASV method. (A) Alpha diversity using inverse-Simpson index of the microbiota between the three conditions in CRC cohort and Non-Cancer subjects. Boxplot represents the inverse-Simpson index across the different groups. (B) Alpha diversity using Chao1 index of the microbiota between the three conditions in CRC cohort and Non-Cancer subjects. The boxplot represents the Chao1 index across the different groups. (A-B) Whiskers represent the minimum and the maximum, while the top, the bottom, and the band in the box represent the first and third quartile and the median respectively. Each data point in the box plot represents one patient, and the large black dot in the middle of each boxplot represents the mean. Shapiro-Wilk normality test demonstrated deviation from normality, so the non-parametric Kruskal-Wallis followed by Post-Hoc Dunn’s test was performed for the statistical comparison between the conditions: * FDR < 0.05, ** FDR < 0.01 and red # FDR for linear mixed effect model accounting for paired samples and with adjustment for age and sex. Further statistical analysis and details were provided in the supplementary methods and supplementary Table 3. (C) Beta Diversity represented as Principal coordinate analysis (PCoA) of microbiota variation in fecal samples collected from Non-Cancer, CRC-Before and CRC-After subjects based on Bray-Curtis distances. (D) Dominant phyla relative abundance for the gut microbiome in all fecal samples of CRC cohort and Non-Cancer subjects. Microbiome data was analyzed using USEARCH ASV method.

Supplementary Material 4: Supplementary Figure 3. Microbiome diversity across three conditions of the CRC cohort and the Non-Cancer subjects using OTU method. (A-C) Alpha diversity using Shannon (A), Inverse Simpson (B), and Chao1 (C) Indices of microbiota between the three conditions in CRC cohort and Non-Cancer subjects. Whiskers represent the minimum and the maximum, while the top, the bottom, and the band in the box represent the first and third quartile and the median respectively. Each data point in the box plot represents one patient, and the large black dot in the middle of each boxplot represents the mean. Shapiro-Wilk normality test demonstrated deviation from normality, so the non-parametric Kruskal-Wallis followed by Post-Hoc Dunn’s test was performed for the statistical comparison between the conditions: * FDR < 0.05, ** FDR < 0.01 and red # FDR for linear mixed effect model accounting for paired samples and with adjustment for age and sex. (D) Beta diversity for the gut microbiome derived from fecal samples of CRC cohort and Non-Cancer subjects. The non-metric multidimensional scaling (NMDS) plot is based on the Bray–Curtis dissimilarity distances. Beta dispersion across the groups was presented as boxplot and differences in community composition between groups were assessed using PERMANOVA. ** FDR < 0.01 and red # p-value for regression model with adjustment for age and sex. (E-F) Dominant phyla relative abundance for the gut microbiome derived from fecal samples of the CRC cohort and Non-Cancer subjects grouped into three conditions (E) or representing all samples (F). (A-F) All graphs include subjects grouped into three conditions: Non-Cancer (*n *= 32), CRC-Before (*n *= 17) meaning CRC patients before therapy and CRC-After (*n *=10) meaning CRC patients after 3-6 months of therapy. Microbiome data was analyzed using USEARCH OTU method.

Supplementary Material 5: Supplementary Figure 4. Comparison of the alpha diversity indices in the CRC cohort and the Non-Cancer subjects using ASV and OTU method. Spearman correlation was used to report the correlation coefficient (r) for ASV and OTU results of the alpha diversity indices.

Supplementary Material 6: Supplementary Figure 5. Differential microbiome abundance across three conditions of the CRC cohort and the Non-Cancer subjects. (A-B) Circo Plot showing the relative abundance of genera and species, respectively, which are significantly differentially abundant in global test when analyzing the three conditions of the CRC cohort and the Non-Cancer subjects without adjustment for age and sex. (C-D) Significant genera and species, respectively, as identified by multiple pairwise comparisons against CRC-Before group (Dunnett’s type of test) without adjustment for age and sex. The columns denote the specific comparisons: CRC-Before versus Non-Cancer and CRC-After versus CRC-Before. The rows list significant genera/species as identified by multiple pairwise comparisons. Each cell color represents significant changes in absolute abundance: purple for increased abundance and pink for reduced abundance. The text within each cell represents the log fold-change value relative to the reference group, with the white color denoting passing the robustness analysis for the pseudo-count addition while the black color denoting not passing it. (E-F) Significant genera and species, respectively, as identified by pattern analysis with Non-Cancer as the reference and without adjustment for age and sex. Monotonically increasing and decreasing trends were evaluated across the three conditions, with Non-Cancer as the reference. The columns denote the three conditions, and the rows contain the significant genera/species identified by pattern analysis. Each cell color represents abundance change: purple for increased abundance and pink for reduced abundance. Each cell contains log fold-change relative to the reference group. Genera/Species names represented in green denote passing the robustness analysis for the pseudo-count addition. (A-F) All graphs include subjects grouped into three conditions: Non-Cancer (*n* = 32), CRC-Before (*n *= 17) meaning CRC patients before therapy and CRC-After (*n *=10) meaning CRC patients after 3-6 months of therapy. Statistical tests were done using ANCOM-BC2 without sex and age adjustment and multiple testing correction for (C-F) was done using the Holm–Bonferroni method.

Supplementary Material 7.

Supplementary Material 8.

## Data Availability

The datasets generated and/or analysed during this study are deposited in the SRA repository under reference PRJNA1427165. A confidential link for reviewers is provided: [https://dataview.ncbi.nlm.nih.gov/object/PRJNA1427165?reviewer=qjt3vivopvr5rhvl2ah37b3gv9]. Datasets will be publicly available upon publication.
