# Supplementary material for: The gut microbiome in the Lebanese population and its alterations in metastatic colorectal cancer: benchmarking and application
Source: BMC Microbiol. 2026 Jun 29;26:809. doi: 10.1186/s12866-026-05325-9 (PMC13579751; doi:10.1186/s12866-026-05325-9)
Supplement: Supplementary file 1 [file 12866_2026_5325_MOESM8_ESM.pdf]

Microbiome Number	
CRC Number	
Patient's Name	
Recruitment Date	
Stool Sample Collection Date	
Storage Temperature	
Storage Location	
Date of FFQ	
Stool Consistency	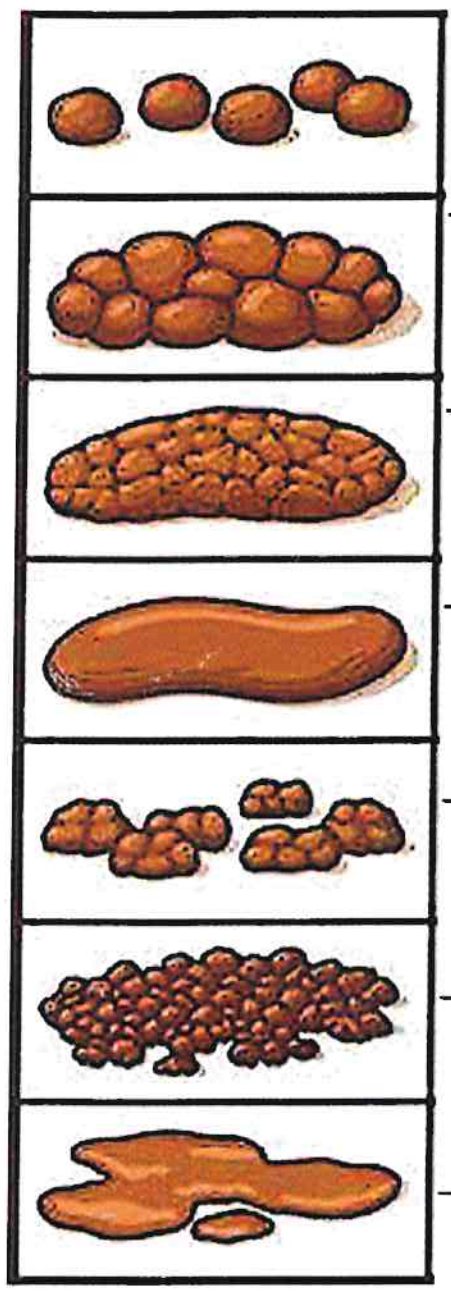

Antibiotics Administration	Name	
	Dose	
	Last Taken	
PPI	Name	
	Dose	
	Last Taken	
Metformin	Last Taken	
NSAIDs	Last Taken	
Probiotics	Last Taken	
Statins	Last Taken	
Chemotherapy Cycle		
Chronic Bowel Disease		
