# Supplementary material for: The gut microbiome in the Lebanese population and its alterations in metastatic colorectal cancer: benchmarking and application
Source: BMC Microbiol. 2026 Jun 29;26:809. doi: 10.1186/s12866-026-05325-9 (PMC13579751; doi:10.1186/s12866-026-05325-9)
Supplement: Supplementary file 2 [file 12866_2026_5325_MOESM1_ESM.docx]

**Supplementary Methods**

**Benchmarking computational tools for microbiome analysis based on mock data**

**1.1 Using Mothur**

The Standard Operation Procedure of Mothur was used as a guideline [46, 47] to analyse 16S rRNA sequencing data (Supplementary Figure 1A). The forward and reverse reads were merged into contigs which were then filtered for the number of ambiguous nucleotides, expected length and the number of homopolymer or repeated nucleotides. Contigs were aligned to a reference to select those mapping to the region of interest. A preliminary clustering which compared contigs to each other and grouped similar sequences took more computing time than other tools which was a factor of exclusion in later analysis on our samples. Chimera, the artifact sequences resulting from the PCR amplification step, were removed after aligning sequences against a chimera reference file (Gold database). Singleton contigs were removed to be consistent with the other pipelines and to reduce computing time. The contigs were aligned to a reference derived from the latest version of Ribosomal Database Project (RDP-18) and clustered using the most efficient method of distance-based greedy clustering ‘dgc’ without the need of forming a distance matrix 20]. A cutoff of 0.03 was selected for the Operational taxonomic unit (OTU) clustering, which translates into clustering to more than 97% of similarity [48]. Finally, the taxonomy was assigned for each cluster.

1.2 **Using USEARCH**

Similar to the Mothur tool, the first steps included merging the reads by aligning the forward and reverse reads and choosing the nucleotide with the best quality score at the overlap region without assigning any ambiguous nucleotide (Supplementary Figure 1B). The default stringent parameters of merging were used for the mock data analysis since both forward and reverse reads were of good quality. The low-quality reads were eliminated by allowing for a maximum expected error of one. Before clustering, sequences were dereplicated and similar sequences were assembled to reduce the computing time. Both OTU clustering using UPARSE algorithm as well as the Amplicon Sequence Variants (ASV) denoising through UNOISE3 were performed along with chimera removal using both algorithms. Finally, taxonomy was assigned by aligning cluster sequences to a reference (RDP-18).

**1.3 Using DADA2**

Unlike the other tools, DADA2 filters and trims both forward and reverse reads before merging them. The DADA2 workflow is adapted from its pipeline available on its website [49] (Supplementary Figure 1C). The most optimized parameters for filtering the reads were selected to perform length based and quality-based trimming as well as to limit the expected number of errors in a read to 1 (similar to the USEARCH pipeline). A model-based approach is used to distinguish between sequencing errors and true biological sequences. This step of the pipeline takes most of the computing time (around 1 minute 30 seconds on the mock sample). The computing time was then reduced by dereplicating the reads and grouping identical sequences. This was followed by a denoising step that assigns biological sequences using the error model. Finally, filtered reads were merged to form contigs. A count table was created that summarizes the abundance of each unique ASV sequence across all samples. Chimeras were removed using a *de novo* algorithm, without the need for a reference database, in contrast with other tools. The taxonomy is assigned using a reference RDP-18 with the minimum number of 80 bootstraps required to return genuine taxonomic classification.

**Statistical power for CRC cohort and Non-Cancer subjects**

To detect a statistically significant difference in all alpha diversity indices between the Non-Cancer and CRC-Before groups, the required sample size per group was estimated to range from 6 to 32, assuming a power of 80% and a significance level (p-value) of 0.05. These calculations were based on the following estimated differences in means (delta means) and corresponding standard deviations (SD) for each alpha diversity index:

- **Shannon index**: Δ mean = 0.7, SD = 0.428
- **Inverse Simpson index**: Δ mean = 4062.8, SD = 2309.67
- **Chao1 index**: Δ mean = 424.8, SD = 476.64

**Our sample sizes are within the required range of 6-32 samples.**

**Statistical Analysis for alpha diversity indices**

Variations in alpha diversity indices (Shannon, Inverse Simpson and Chao1) were assessed with respect to age, sex, BMI and across the three conditions (Non-Cancer, CRC-Before, and CRC-After) for both ASV and OTU analyses (Supplementary Table 3). Associations with age, sex and BMI were assessed using Linear Mixed Effect Model (LMM) which accounts for repeated measures (Supplementary Table 3a, 3c and 3e). To evaluate associations with the three conditions, two complementary statistical frameworks were applied to ensure robustness. First, the non-parametric Kruskal-Wallis test, followed by the post hoc Dunn’s test with FDR correction, was performed for comparisons across the three conditions, given the deviation of the alpha diversity indices from normality as per the normality testing (Supplementary Table 3b and 3d). However, as Kruskal-Wallis test considers the three conditions independent and does not allow covariate adjustment, a second statistical test was conducted using both crude and age/sex-adjusted LMMs with an assumption of normality since the skewness values were either approximately symmetric or only moderately skewed (Supplementary Table 3b, 3d and 3f). Covariate adjustment was performed only for age and sex as both showed borderline significance with alpha diversity indices, while BMI showed no significant association with any index (Table 2). Results were quite concordant across the various statistical approaches (Supplementary Table 3). Finally, Spearman correlation analysis was performed to compare alpha diversity indices from the OTU and ASV method, revealing consistent positive correlations across methods (Supplementary Figure 4).
